# Supplementary material for: Gut microbiome dysbiosis drives metabolic dysfunction in Familial dysautonomia
Source: Nat Commun. 2023 Jan 13;14:218. doi: 10.1038/s41467-023-35787-8 (PMC9839693; doi:10.1038/s41467-023-35787-8)
Supplement: Supplementary file 2 — Description of Additional Supplementary Files [file 41467_2023_35787_MOESM2_ESM.pdf]

File Name: Supplementary Data 1

Description: This is a Microsoft Excel file containing Supplementary Tables 1a and 1b and Supplementary Table 2 as separate tabs. Supplementary Table 1a contains de-identified metadata on FD patients and paired relatives and identifies those used to generate individual ordinations in panels of Figure 1a, e, and f in the main text. Similarly, Supplementary Table 1b identifies FD patient-relative pairs used for microbiome diversity analyses (Fig. 1c and d) and metabolome diversity analyses (Fig. 2b, c, and d) in the main text. Metadata for FD patients shown in Supplementary Table 1a was also used to test for correlations between patient factors and microbiome/metabolome diversity shown in Supplementary Table 2 (see footnotes).
