# Supplementary material for: Gut microbiome dysbiosis drives metabolic dysfunction in Familial dysautonomia
Source: Nat Commun. 2023 Jan 13;14:218. doi: 10.1038/s41467-023-35787-8 (PMC9839693; doi:10.1038/s41467-023-35787-8)
Supplement: Supplementary file 4 — Reporting Summary [file 41467_2023_35787_MOESM4_ESM.pdf]

nature portfolio

Corresponding author(s): Frances Lefcort
Valerie Copie
Seth Walk

Last updated by author(s): Dec 6, 2022

Reporting Summary

Nature Portfolio wishes to improve the reproducibility of the work that we publish. This form provides structure for consistency and transparency in reporting. For further information on Nature Portfolio policies, see our Editorial Policies and the Editorial Policy Checklist.

Statistics

For all statistical analyses, confirm that the following items are present in the figure legend, table legend, main text, or Methods section.

n/a	Confirmed	
☐	☒	The exact sample size (*n*) for each experimental group/condition, given as a discrete number and unit of measurement
☐	☒	A statement on whether measurements were taken from distinct samples or whether the same sample was measured repeatedly
☐	☒	The statistical test(s) used AND whether they are one- or two-sided<br>*Only common tests should be described solely by name; describe more complex techniques in the Methods section.*
☐	☒	A description of all covariates tested
☐	☒	A description of any assumptions or corrections, such as tests of normality and adjustment for multiple comparisons
☐	☒	A full description of the statistical parameters including central tendency (e.g. means) or other basic estimates (e.g. regression coefficient) AND variation (e.g. standard deviation) or associated estimates of uncertainty (e.g. confidence intervals)
☐	☒	For null hypothesis testing, the test statistic (e.g. *F*, *t*, *r*) with confidence intervals, effect sizes, degrees of freedom and *P* value noted<br>*Give P values as exact values whenever suitable.*
☒	☐	For Bayesian analysis, information on the choice of priors and Markov chain Monte Carlo settings
☒	☐	For hierarchical and complex designs, identification of the appropriate level for tests and full reporting of outcomes
☐	☒	Estimates of effect sizes (e.g. Cohen's *d*, Pearson's *r*), indicating how they were calculated

Our web collection on statistics for biologists contains articles on many of the points above.

Software and code

Policy information about availability of computer code

Data collection	No computer programs or software was used to collect data in this study.
Data analysis	Chenomx NMR Suite software (version 8.4), MetaboAnalyst (version 3.0), Progenesis QI (version 3.0), Topspin (Bruker version 3.2), mothur (version 1.46.1), VSEARCH, and R (version 4.1.0 with the following packages: vegan, version 2.6-2; vegan3d, version 1.1-2; labdsv, version 2.0-1; randomForest, version 4.7-1; rfutilities, version 2.1-5; cluster, version 2.1.3; vioplot, version 0.3.7; lme4, version 1.1-29; gplots, version 3.1.3; RcolorBrewer 1.1-3; and dyplr, version 1.0.10; and mixOmics version 6.16.3) were used for data analysis as described in the Methods section. Custom scripts were used to perform permutation analysis as described and are publicly available (https://github.com/nvpinkham/Dysautonomia) and linked to the Zenodo repository (http://dx.doi.org/10.5281/zenodo.7384390).

For manuscripts utilizing custom algorithms or software that are central to the research but not yet described in published literature, software must be made available to editors and reviewers. We strongly encourage code deposition in a community repository (e.g. GitHub). See the Nature Portfolio guidelines for submitting code & software for further information.

Data

Policy information about availability of data

All manuscripts must include a data availability statement. This statement should provide the following information, where applicable:

- Accession codes, unique identifiers, or web links for publicly available datasets
- A description of any restrictions on data availability
- For clinical datasets or third party data, please ensure that the statement adheres to our policy

All sequencing reads generated in this study have been deposited in the National Center for Biotechnology Information (NCBI) BioProject database under accession number PRJNA785599 (https://www.ncbi.nlm.nih.gov/bioproject/?term=PRJNA785599). All metabolomics data generated in this study have been deposited in the MetaboLights repository under accession number MTBLS5138 (https://www.ebi.ac.uk/metabolights/MTBLS5138) and are also available at GitHub (https://github.com/nvpinkham/Dysautonomia). The data and code used for analyses generated in this study are available as a single compressed Source Data file on GitHub (https://github.com/nvpinkham/Dysautonomia) and deposited at the Zenodo repository (http://dx.doi.org/10.5281/zenodo.7384390). The Silva 16S rRNA database used for alignment is available at https://www.arb-silva.de/documentation/release-128 and the RDP training set used is available at https://sourceforge.net/projects/rdp-classifier/files/RDP_Classifier_TrainingData. Microbiome (16S rRNA sequencing) datasets used for comparisons are available at the NCBI BioProject database accession numbers PRJNA505353 (Martinson et al.) and PRJNA290926 (Baxter et al.).

Human research participants

Policy information about studies involving human research participants and Sex and Gender in Research.

Reporting on sex and gender	Both sexes were included but not collected or considered in a priori comparisons. We felt this design was reasonably justified given the small number of individuals with FD (n~350) worldwide). No information regarding gender was collected or considered.
Population characteristics	Several covariates were considered in the study and are reported in Supplementary Data Table 1 (weight, BMI, age, ability to eat food orally, antibiotic usage in the previous 3 months, and a clinical chemistry panel).
Recruitment	Patients were approached at annual clinic visits to NYU Langone Medical Center. If interested, cohabitating relatives were also approached about the study. All subjects (patients and relatives) were recruited with informed consent following IRB-approved protocols. The potential for self-selection bias was deemed minimal due to the genetic predisposition and highly penetrant nature of the disease. The Dysautonomia Center at NYU Langone serves the majority of FD patients around the world and given the prevalence of the disease in the Ashkenazi Jewish community, we assumed minimal potential of bias due to sample size.
Ethics oversight	Samples were obtained from enrolled participants under Institutional Review Board approved protocols at both Montana State University and the New York University School of Medicine (NYU Langone Health).

Note that full information on the approval of the study protocol must also be provided in the manuscript.

Field-specific reporting

Please select the one below that is the best fit for your research. If you are not sure, read the appropriate sections before making your selection.

[x] Life sciences [ ] Behavioural & social sciences [ ] Ecological, evolutionary & environmental sciences

For a reference copy of the document with all sections, see nature.com/documents/nr-reporting-summary-flat.pdf

Life sciences study design

All studies must disclose on these points even when the disclosure is negative.

Sample size	No sample size calculations were performed a priori. As many samples from human subjects were included as possible using analyses that accounted for multiple samples from some (but not all) subjects. Mouse experiments were typically based on a minimum of three experimental animals per cage, and included biological replicates (multiple cages) to add further support for experimental treatment effects.
Data exclusions	Samples were excluded from paired, patient-relative comparisons if collected more than 90 days apart. Some 16S sequencing reads were filtered from the dataset due primarily to poor quality, incorrect length, and when evidence of a chimeric sequence was found.
Replication	As described in the manuscript, replication was used in mouse experiments to confirm treatment effects. Figures include more than one group of experimental animals that served as independent, biological replicates.
Randomization	For experiments with human subjects, individuals were allocated to the patient cohort based on genotype (homozygous for the FD-causing mutation in ELP1) and presentation to the FD Dysautonomia Center for annual clinic visits. Paired, cohabitating relatives were also identified at clinic visits. Covariates were not controlled during allocation. Rather, potential covariates were included in analyses as mixed effects. Experimental animals were randomized to cages based on sex (males and females housed separately) and genotype (FD and control mice, as

	described). In some cases, randomization was not possible, as all mice in a particular litter were needed to achieve the desired sample size.
Blinding	No blinding was done in this study. As per local regulatory guidelines, treatment groups in animal experiments had to be clearly labeled in order to assess animal health and welfare, and to ensure appropriate exposure conditions.

Reporting for specific materials, systems and methods

We require information from authors about some types of materials, experimental systems and methods used in many studies. Here, indicate whether each material, system or method listed is relevant to your study. If you are not sure if a list item applies to your research, read the appropriate section before selecting a response.

Materials & experimental systems

n/a	Involved in the study
☒	☐ Antibodies
☒	☐ Eukaryotic cell lines
☒	☐ Palaeontology and archaeology
☐	☒ Animals and other organisms
☒	☐ Clinical data
☒	☐ Dual use research of concern

Methods

n/a	Involved in the study
☒	☐ ChIP-seq
☒	☐ Flow cytometry
☒	☐ MRI-based neuroimaging

Animals and other research organisms

Policy information about studies involving animals; ARRIVE guidelines recommended for reporting animal research, and Sex and Gender in Research

Laboratory animals	Mus musculus, C57BL/6, specific pathogen free (SPF), Tuba1a-Cre+; Elp1loxp/loxp (FD mice), Tuba1a-Cre-; /Elp1+/loxp (control mice) were used. Ages ranged from 3-11 months in age; 21 days (0 days post-weaning; DPW) to 485 days (465 DPW).
Wild animals	No wild animals were used in this study.
Reporting on sex	Both sexes were used but sex was not considered in the study design. The sex of pups was determined at weaning based on handling and visual inspection.
Field-collected samples	No field-collected samples were used in this study.
Ethics oversight	All mouse experiments were conducted in the AALAC-accredited Animal Resource Center at Montana State University under local IACUC-approved protocols.

Note that full information on the approval of the study protocol must also be provided in the manuscript.
